# The efficacy of fascia iliaca compartment block for pain control after total hip arthroplasty: a meta-analysis

**DOI:** 10.1186/s13018-018-1053-1

**Published:** 2019-01-25

**Authors:** Xiao-yan Zhang, Jian-bao Ma

**Affiliations:** Department of Anesthesiology, Linyi people’s Hospital, No. 49 Yizhou Road, Lanshan District, Linyi, 276003 Shandong China

**Keywords:** Fascia iliaca compartment block, Total hip arthroplasty, Meta-analysis

## Abstract

**Purpose:**

Fascia iliaca compartment block (FICB) provides an analgesic option for total hip arthroplasty (THA) patients. The evidence supporting FICB is still not well established. The purpose of this meta-analysis was to assess FICB for pain control in THA patients.

**Methods:**

PubMed, Embase, Cochrane Library, and Chinese Wanfang database were interrogated from their inceptions to December 15, 2018. We included randomized controlled studies reported as full text, those published as abstracts only, and unpublished data, if available. Data were independently extracted by two reviewers and synthesized using a random-effects model or fixed-effects model according to the heterogeneity.

**Results:**

A total of eight RCTs were finally included for meta-analysis. Compared with placebo, FICB could significantly reduce VAS pain scores at 1–8 h (WMD = − 0.78, 95% CI [− 1.01, − 0.56], *P* = 0.000), 12 h (WMD = − 0.69, 95% CI [− 1.22, − 0.16], *P* = 0.011), and 24 h (WMD = − 0.46, 95% CI [− 0.89, − 0.02], *P* = 0.039). Compared with the control group, FICB could significantly decrease the occurrence of nausea and length of hospital stay (*P* < 0.05). There was no significant difference between the VAS pain score at 48 h and risk of fall between the FICB and the control groups (*P* > 0.05).

**Conclusions:**

FICB could be used to effectively reduce pain intensity up to 24 h, total morphine consumption, and length of hospital stay in THA patients. Optimal strategies of FICB need to be studied in the future.

**Electronic supplementary material:**

The online version of this article (10.1186/s13018-018-1053-1) contains supplementary material, which is available to authorized users.

## Introduction

Total hip arthroplasty (THA) is a well-known popular surgical procedure for hip diseases, including end-stage hip osteoarthritis and femur neck fracture [1, 2]. THA is a well-known cause of severe postoperative pain. Non-steroidal antiinflammatory drugs (NSAIDs) and morphine were commonly used for pain control in THA patients [2]. These drugs were related to digestive tract side effects, and patients could not tolerate these complications [3, 4]. Thus, adequate pain control after THA is crucial for early ambulation and patient satisfaction.

Fascia iliaca compartment block (FICB) is an analgesic technique that involves injecting local anesthetic under the fascia of the iliacus muscle [5, 6]. FICB can be performed either guided by ultrasound or with a loss of resistance (LOR) technique. The evidence supporting FICB for pain control for THA patients is still not well established. Several RCTs have been published concerning FICB for THA patients. However, there was no consensus about the real efficacy of FICB in THA patients. Desmet et al. [7] revealed that longitudinal suprainguinal FICB reduces postoperative morphine requirements after anterior approach THA. Perry et al. [8] found that FICB has comparable pain control with psoas compartment block. The samples of published literature are limited, and the statistic power is limited. However, Aprato et al. [9] revealed that intra-articular hip injection provides better pre-operatory pain management in elder patients with intracapsular hip fractures compared to the FICB.

Therefore, we performed a meta-analysis of randomized controlled trials to compare the effect of FICB versus control on the pain intensity and morphine consumption in patients undergoing THA. We hypothesized that FICB compared with placebo is associated with decreased pain intensity in patients undergoing THA. Additionally, we assessed the efficacy and safety of FICB with respect to morphine consumption, risk of fall, and nausea.

## Materials and methods

This meta-analysis was programed on the basis of the Preferred Reporting Items for Systematic Reviews and Meta-Analyses (PRISMA) statement guidelines [10]. There was no registered protocol for this meta-analysis.

### Search strategy

We performed a systematic electronic search in PubMed, Embase, the Cochrane library, and Chinese Wanfang database from inception through September 1, 2018. We conducted electronic searches using exploded Medical Subject Headings (MeSH) terms and corresponding key words. The search terms used in PubMed were listed in Additional file 1. No language restriction was applied. We also manually checked the bibliographies of previous reviews and included trials to identify other potentially eligible trials.

### Inclusion criteria

Published RCTs meeting the following criteria were included: (1) population: adult patients and prepared for THA, (2) intervention: single administration FICB for pain control, (3) comparison: placebo or saline, and (4) ≥ 1 of the following outcomes: visual analog scale (VAS) at 6–8 h, 12 h, 24 h, and 48 h; total morphine consumption; occurrence of nausea; and occurrence of fall.

### Study selection

After the duplicates were removed and the study selection process was completed, the titles and abstracts were scanned by two independent investigators. The relevant data were extracted by adopting a predetermined standardized procedure, which involved the first authors, year of publication, country, demographic characteristics of the participants, and the treatment regimen for each group. All data were verified for internal consistency, and controversies were settled by consensus or discussion with a third author. When inadequate information existed in the studies, contacting the first authors to obtain and clarify the relevant data were essential as specified by the standardized protocol.

### Quality assessment

Cochrane collaboration’s tool for assessing the risk of bias was used to evaluate methodological quality of included trials. This tool focuses on the internal validity of the trial and assessment of risk of possible bias in different phases of trial conduct. The following items were assessed: random sequence generation, allocation concealment, blinding of participants, personnel and outcome assessment, incomplete outcome measures, selective outcome reporting, and other types of bias. Each item was qualified as low risk (L), unclear risk (U), or high risk (H). All assessments were conducted by two reviewers, independent of each other. Controversies were settled by consensus or discussion with a third author.

### Data extraction

Two authors independently extracted the information from the original studies using a standardized data abstraction list, including study characteristics (such as author, publication year, country), patient characteristics (such as number of patients, mean age, gender, and female patients), intervention details for each treatment group (intervention type, dose, drugs and regimens), and outcome measures (VAS at 6–8 h, 12 h, 24 h, 48 h; total morphine consumption; occurrence of nausea; and occurrence of fall). Data were abstracted from the article text, tables, and graphs.

### Data synthesis and statistical methods

In this meta-analysis, effect sizes for dichotomous outcomes were expressed as relative risk (RR) with 95% confidence interval (CI). Mean difference (MD) and 95% CI were calculated for continuous outcomes. The effect sizes were computed by a random-effects model [11]. *I*^2^ statistic was used to estimate the heterogeneity, with values greater than 50% considered as significant heterogeneity [12]. Furthermore, we applied sensitivity analyses to verify the robustness of the study results by using removing trials one by one. Egger linear regression test and funnel plots were implemented to test the publication bias when more than ten publications were included [13]. *P* values < 0.05 denoted statistically significant differences. Data analysis was conducted with Stata 12.0 (Stata Corp., College Station, TX, USA).

## Results

### Search results

**Fig. 1:**
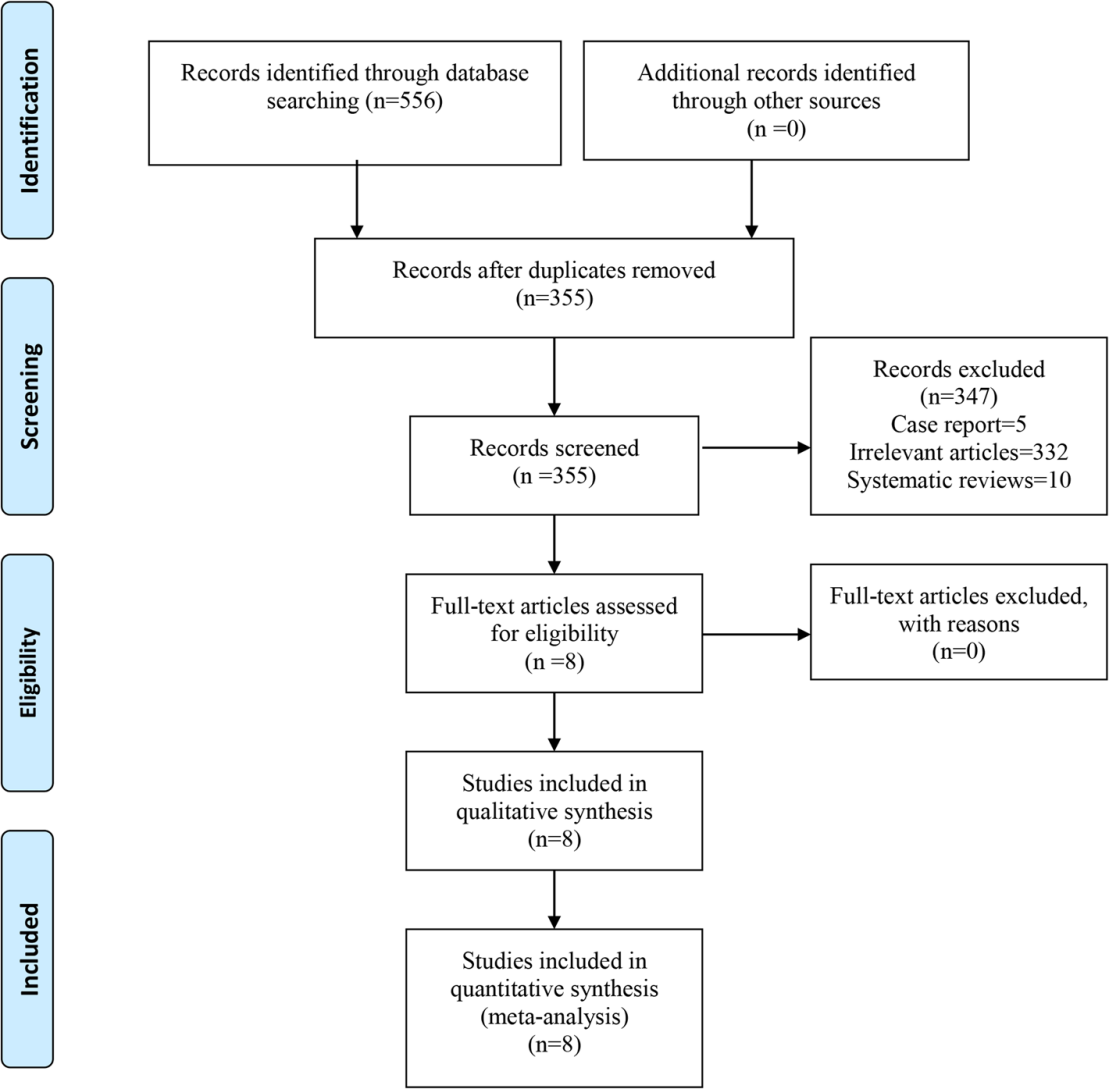
Flow diagram of the study selection process

The PRISMA statement flowchart shows the process of literature screening, study selection, and reasons for exclusion (Fig. 1). Our initial search yielded 556 potential studies. After removing duplicates by Endnote Software (Version X7, Thompson Reuters, CA, USA) and screening the titles and abstracts, eight RCTs were included in this meta-analysis [7, 14–20].

### General characteristic

Characteristics and demographics of the included studies were presented in Table 1. These trials were published from 2009 to 2017. Population sizes ranged from 11 to 43, with a total of 372 patients (FICB = 186, control = 186). In the included studies, age ranged from 54 to 71.2 years. Protocol of FICB included ropivacaine and bupivacaine combined with or no epinephrine. Dose of infiltration drugs ranged from 30 to 50 ml. Only one study reported complication (hematoma) about FICB. Most of the included studies were performed with a relative short follow-up.

**Table 1:** General characteristic of the included studies. FICB, fascia iliaca compartment block

Author	Country	Size (*n*)	Age (years)	Female (%)	Intervention	Control	Nature of study	Complications	Technique	Anesthesia	Follow-up
Shariat 2013	USA	16/16	61.0	56.2	30 mL 0.5% ropivacaine	Placebo	RCT	NS	Parallel	GA	NS
Stevens 2007	Australia	22/22	54.2	50.0	40 mL mixture of 30 mL of 0.5% bupivacaine with 1:200000 epinephrine and 150 μg of clonidine	Placebo	RCT	NS	Perpendicular	SA	At discharge
Desmet 2017	Belgium	43/42	67.5	65.4	40 mL of ropivacaine 0.5%	Placebo	RCT	NS	Perpendicular	GA	6 months
Bang 2016	Korea	11/11	81.6	85.2	40 mL of ropivacaine 0.2% with epinephrine 5 mg/mL	Placebo	RCT	NS	Parallel	GA	NS
Goitia 2009	Spanish	21/20	55.6	55.0	40 ml of 5% bupivacaine	Placebo	RCT	NS	Parallel	NS	NS
Deniz 2014	Turkey	20/20	59.1	56.5	2% prilocaine, 30 ml of 0.25% bupivacaine (1 mg/kg)	Placebo	RCT	NS	Perpendicular	GA	3 days
Cucereanu Badica 2010	Romania	30/ 32	71.0	35.4	50 mL of ropivacaine 0.2% with epinephrine	Placebo	RCT	NS	Perpendicular	GA	NS
Lei 2016	China	23/23	68.7	45.2	40 ml of 5% bupivacaine	Placebo	RCT	Hematoma (*n* = 1)	Parallel	GA	2 days

### Risk of bias

**Fig. 2:**
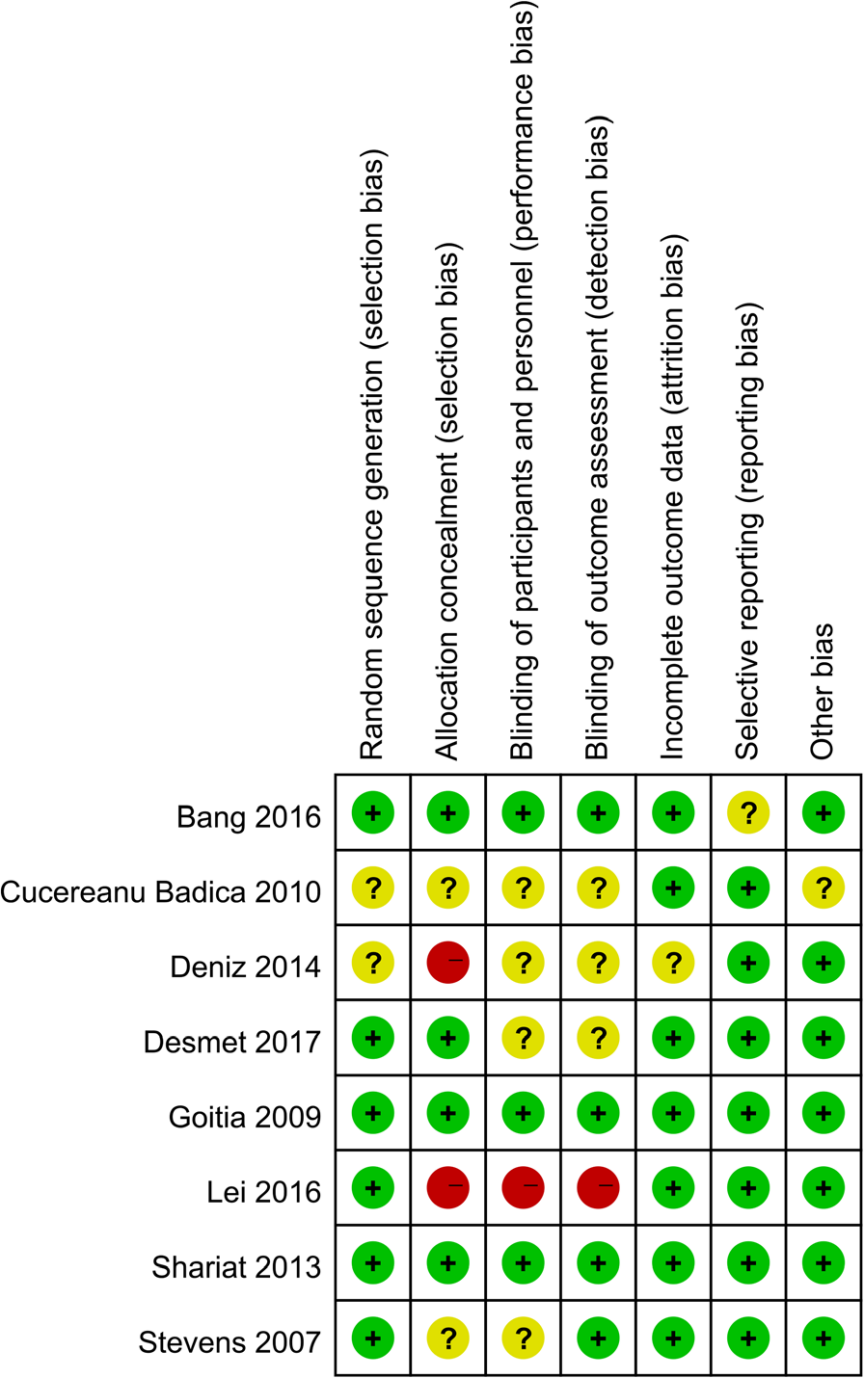
Risk of bias summary

**Fig. 3:**
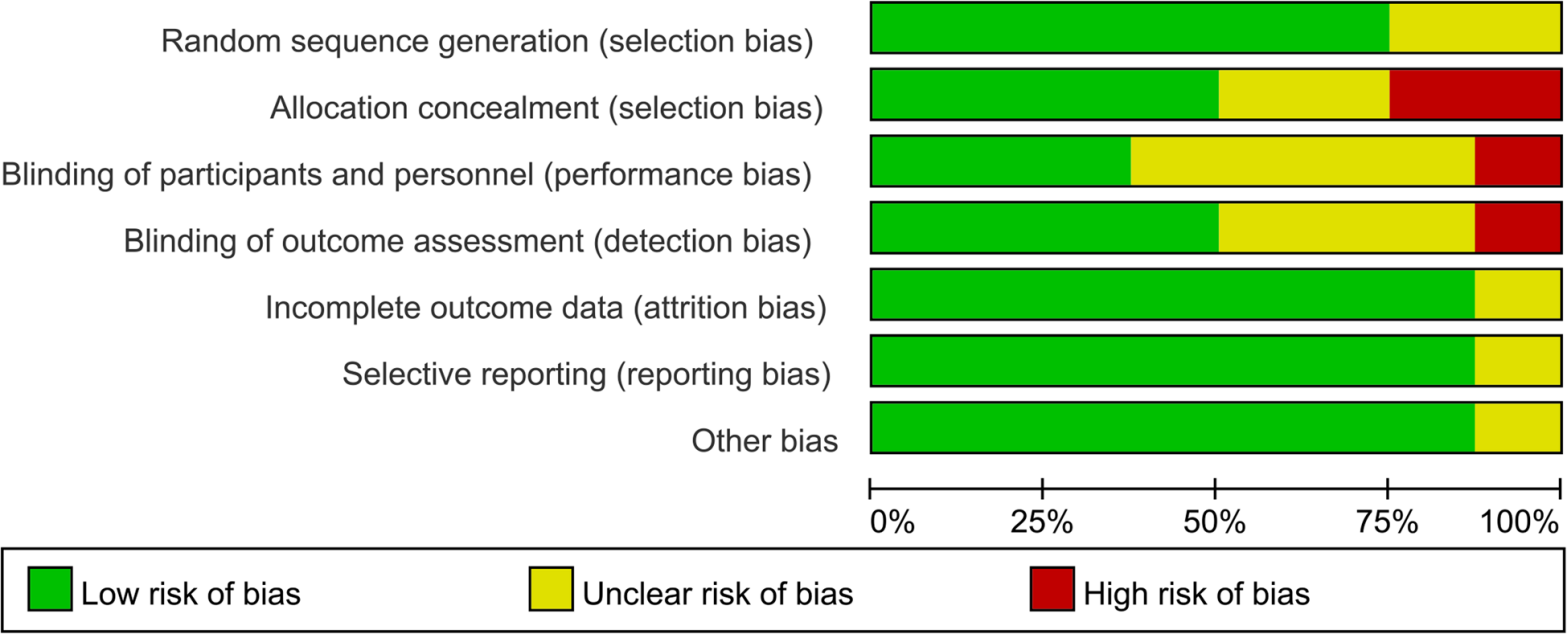
Risk of bias graph

Figures 2 and 3 describe the risk of bias summary and risk of bias graph, respectively. Overall, two trials were categorized as being at low risk of bias, four as being unclear, and two as being at high risk of bias.

### Results of meta-analysis

#### VAS at 1–8 h, 12 h, 24 h, and 48 h

**Fig. 4:**
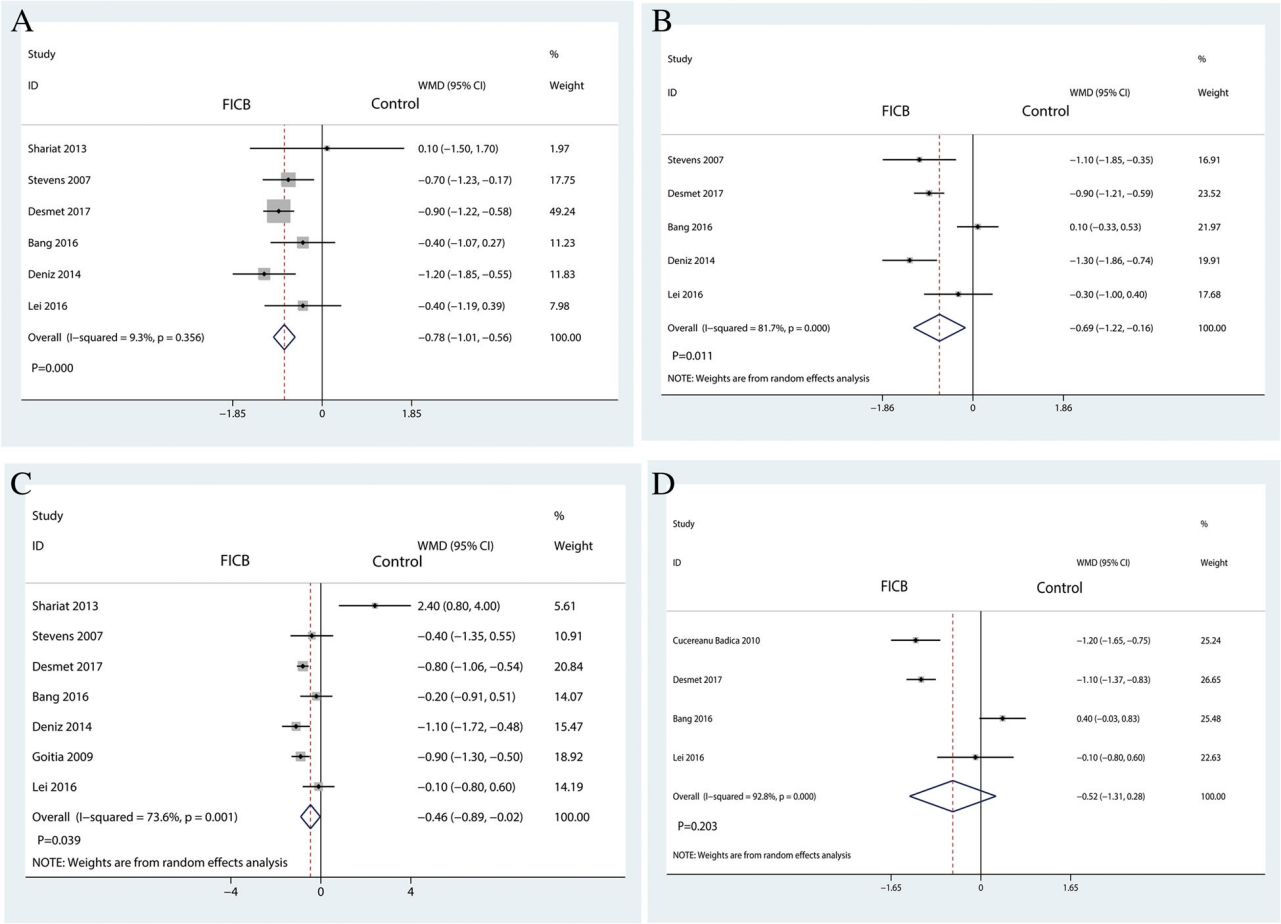
Forest plot for the comparison of VAS at 1–8 h (**a**), VAS at 12 h (**b**), VAS at 24 h (**c**), and VAS at 48 h (**d**) between the FICB group and the control group

The VAS pain scores after treatment for 1–8 h weeks were reported in four trials. There was a little heterogeneity between the included studies (*I*^2^ = 9.3%, *P* = 0.356); fixed-effects model was adapted to analyze the results. The result revealed that that FICB was superior to control in terms of VAS pain scores after intervention for 1–8 h (WMD = − 0.78, 95% CI [− 1.01, − 0.56], *P* = 0.000, Fig. 4a). There was a large heterogeneity between the included studies for VAS pain scores after treatment for 12 h (*I*^2^ = 81.7%, *P* = 0.000, Fig. 4b), 24 h (*I*^2^ = 73.6%, *P* = 0.001, Fig. 4c), and 48 h (*I*^2^ = 92.8%, *P* = 0.000, Fig. 4d). Random-effects model was performed for VAS pain score at 12 h, 24 h, and 48 h. Results have shown that FICB has a beneficial role in reducing VAS pain scores at 12 h (WMD = − 0.69, 95% CI [− 1.22, − 0.16], *P* = 0.011, Fig. 4b) and 24 h (WMD = − 0.46, 95% CI [− 0.89, − 0.02], *P* = 0.039, Fig. 4c). Pooled data found that no statistically significant difference was observed between the FICB group and the control group in terms of the VAS scores after treatment for 72 h (WMD = − 0.52, 95% CI [− 1.31, 0.28], *P* = 0.203, Fig. 4d).

#### Total morphine consumption

**Fig. 5:**
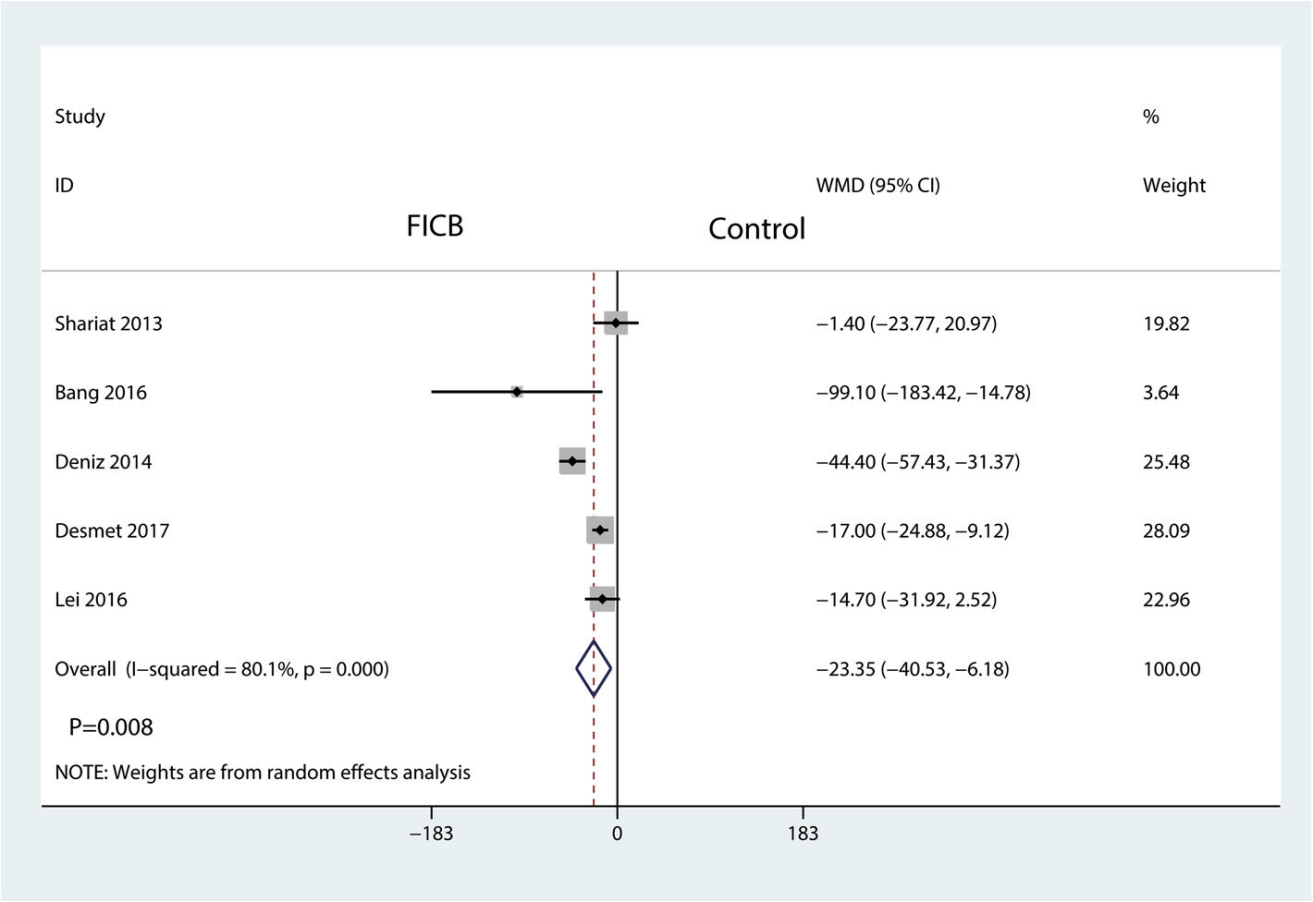
Forest plot for the comparison of total morphine consumption between the FICB group and the control group

A total of five studies reported total morphine consumption between the FICB group and the control group. We applied a random-effects model to analyze the results since there was a large heterogeneity between the included studies (*I*^2^ = 80.01%, *P* = 0.000). The result shows that the FICB could significantly reduce total morphine consumption than the control group (WMD = − 23.35, 95% CI [− 40.53, − 6.18], *P* = 0.008, Fig. 5).

#### Length of hospital stay

**Fig. 6:**
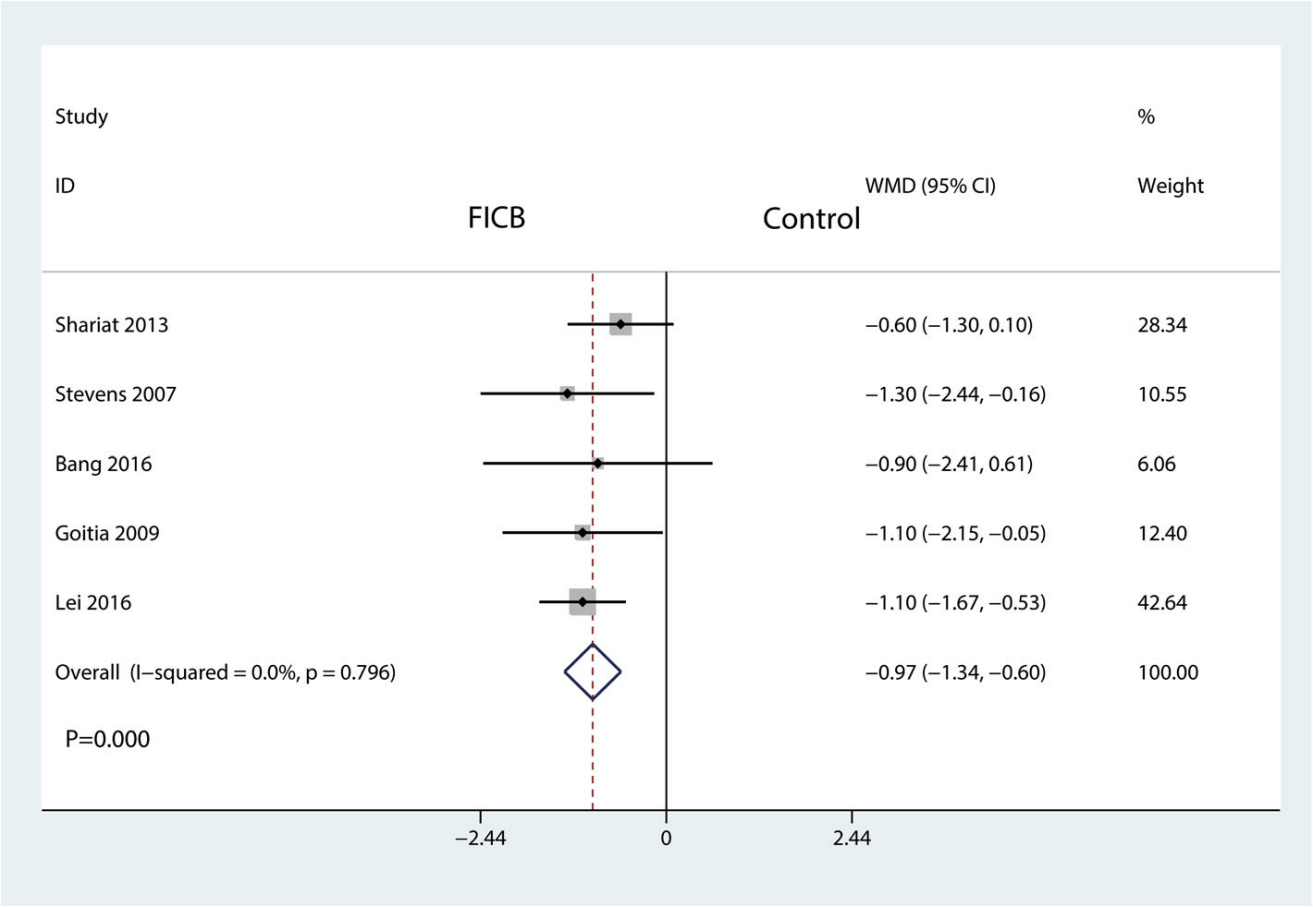
Forest plot for the comparison of the length of hospital stay between the FICB group and the control group

A total of five studies reported the length of hospital stay between the FICB group and the control group. We applied a fixed-effects model to analyze the results since there was no heterogeneity between the included studies (*I*^2^ = 0.0%, *P* = 0.796). The result shows that the FICB could significantly reduce the length of hospital stay than the control group (WMD = − 0.97, 95% CI [− 1.34, − 0.60], *P* = 0.000, Fig. 6).

#### The occurrence of nausea

**Fig. 7:**
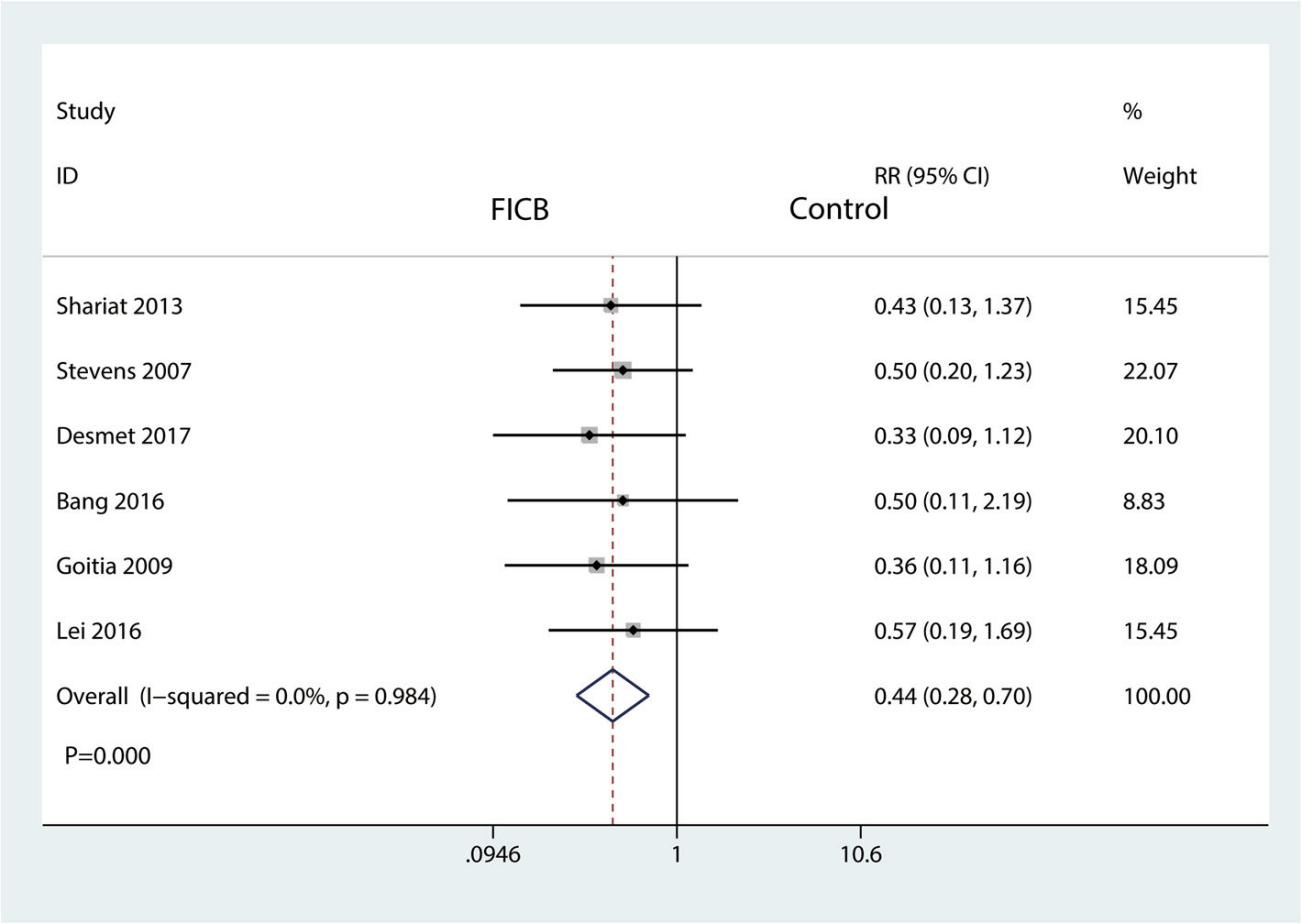
Forest plot for the comparison of risk of nausea between the FICB group and the control group

Six studies involving 179 patients were available for analysis of the occurrence of nausea. The FICB led to significantly less occurrence of nausea than the control group (RR = 0.44, 95% CI [0.28 to 0.70], *P* = 0.000; *I*^2^ = 0.0%, *P* = 0.984, Fig. 7). Thus, we used a fixed-effects model to pool the relevant data.

#### The occurrence of fall

**Fig. 8:**
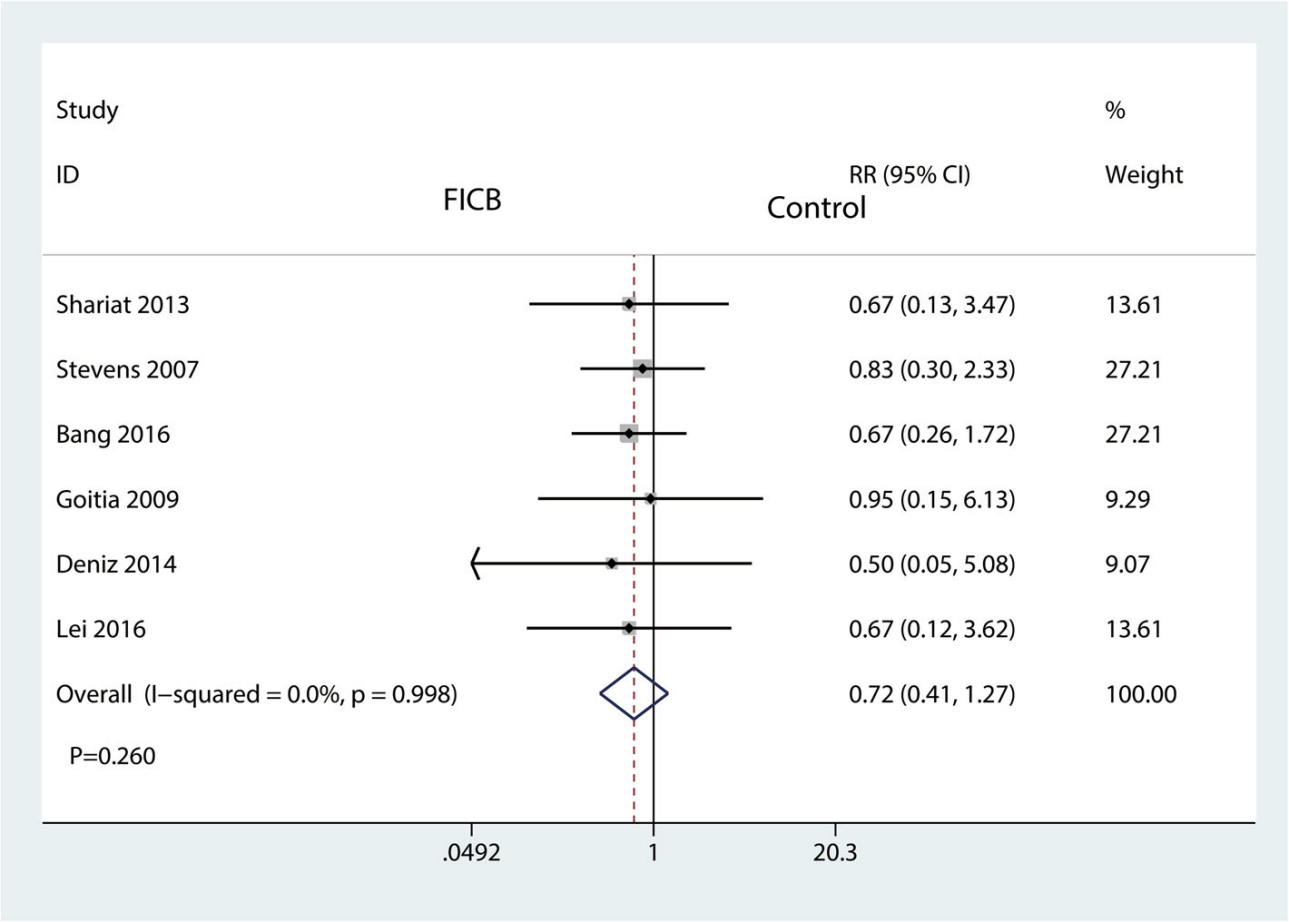
Forest plot for the comparison of the occurrence of fall between the FICB group and the control group

Six studies provided data on the occurrence of fall. FICB was not associated with an increase of the occurrence of fall (RR = 0.72, 95% CI [0.41 to 1.27], *P* = 0.260 Fig. 8). There was no heterogeneity between the included studies (*I*^2^ = 0.0%, *P* = 0.998); thus, we used a fixed-effects model to pool the occurrence of fall.

#### Publication bias, sensitivity analysis, and subgroup analysis

**Fig. 9:**
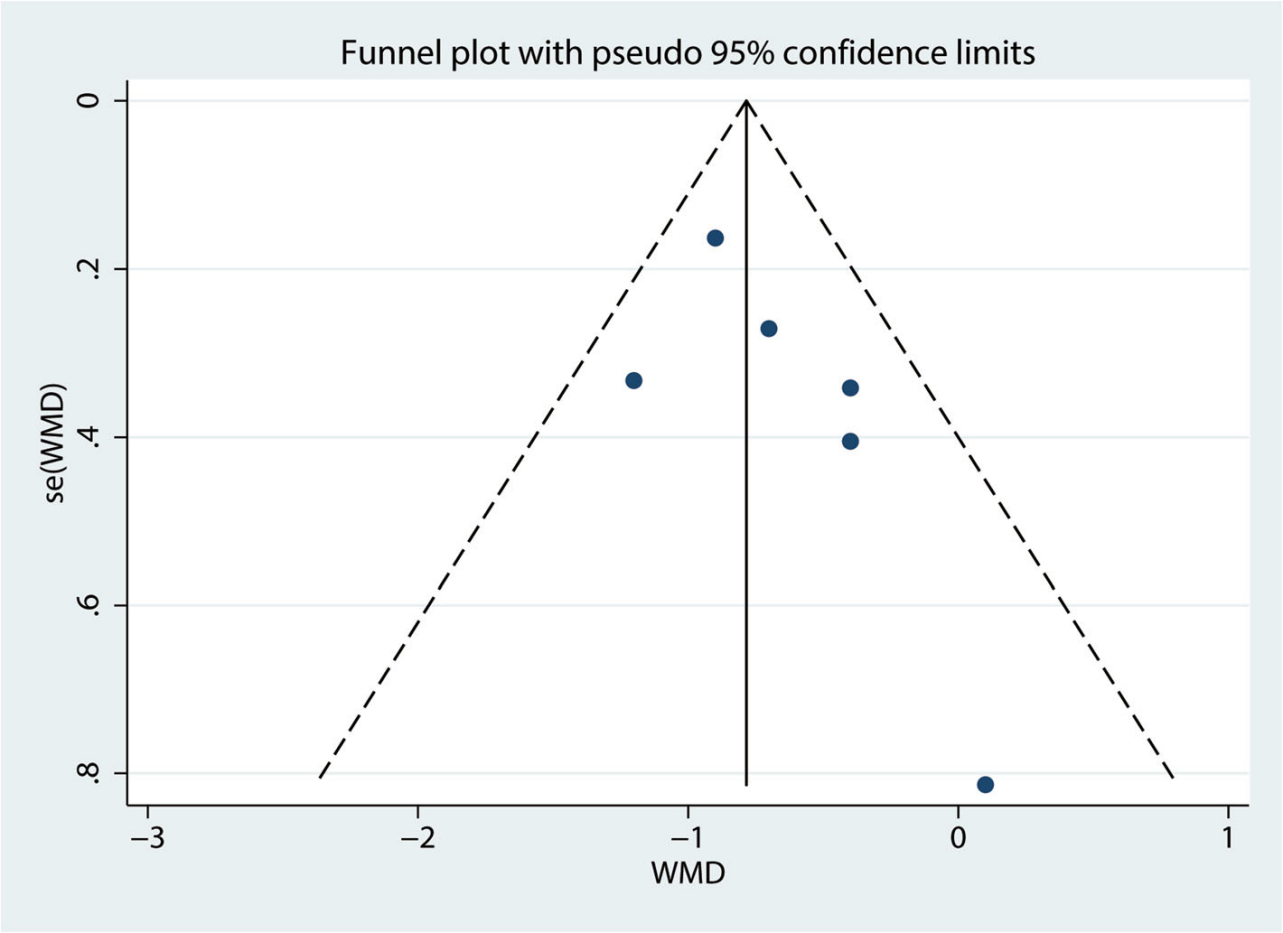
Funnel plot for the comparison of the VAS at 1–8 h between the FICB group and the control group

**Fig. 10:**
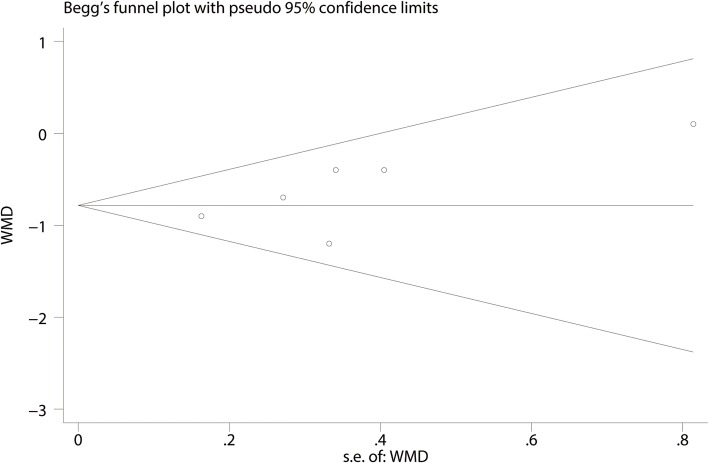
Begg’s test for the comparison of the VAS at 1–8 h between the FICB group and the control group

The funnel plot (Fig. 9) and Begg’s test (Fig. 10) showed no publication bias in the included studies.

**Fig. 11:**
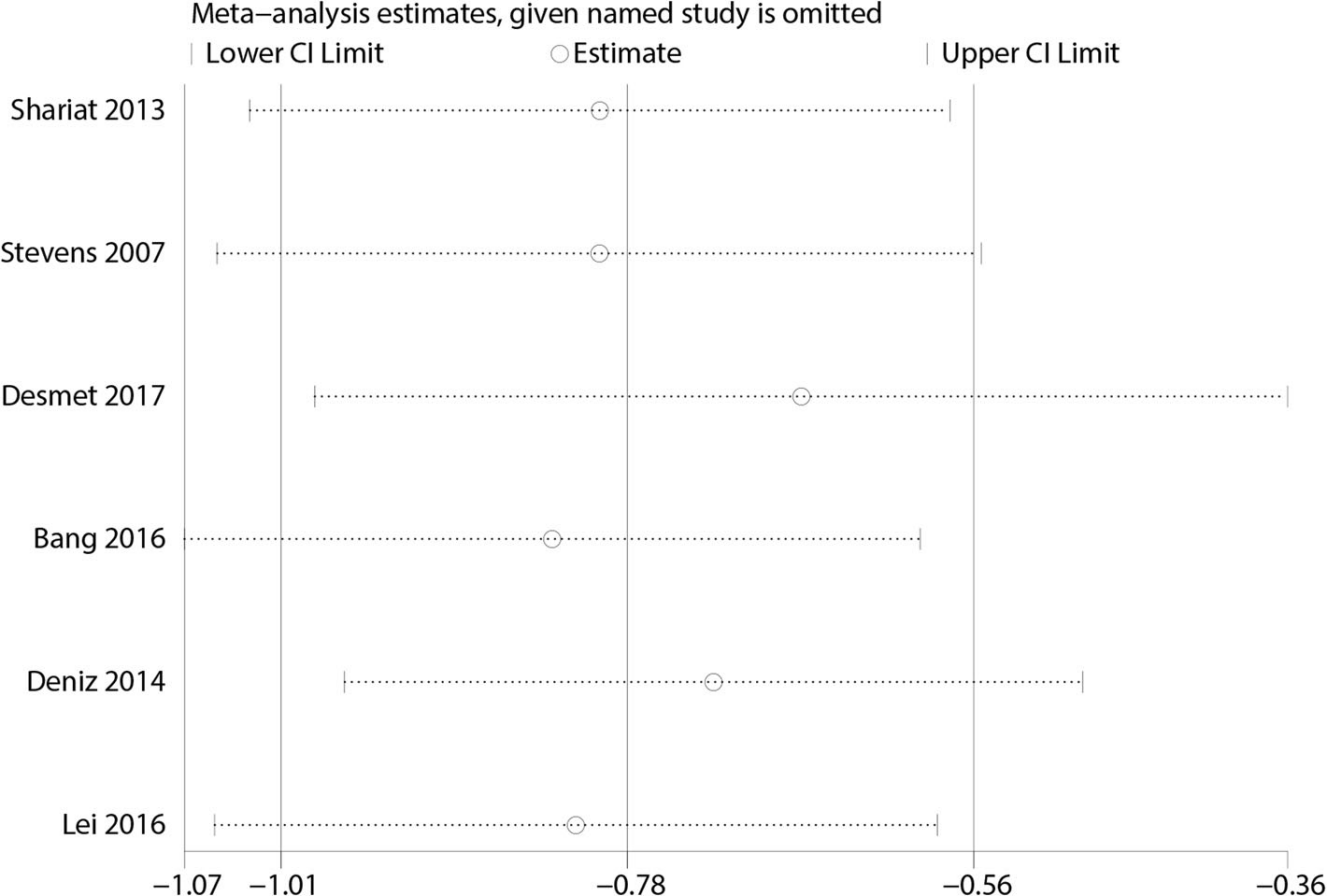
Sensitivity analysis of the VAS at 6–8 h between the FICB group and the control group

Sensitivity analysis was performed by excluding one trail in turn and recalculating the pooled WMD for the remaining trials, which found that none of the studies affected the result (Fig. 11).

## Discussion

### Principal findings

The pooled results indicate that, according to available evidence, THA patient administration with FICB was associated with a reduction of pain intensity up to 24 h. What is more, FICB could significantly reduce total morphine consumption and the risk of nausea. However, there was no significant difference between the FICB and the control in the pain intensity at 48 h and risk of fall.

### Relation to other systematic reviews

There were two meta-analyses comparing FICB and placebo that have been published [21, 22]. Zhang et al. [21] found that FICB has a beneficial role in controlling pain in total hip and knee arthroplasties. Limitation of this meta-analysis was obvious; THA and total knee arthroplasty were two different surgeries, and thus, pain intensity was different. Fei et al. [22] conducted a meta-analysis about FICB for pain control in lower limb surgery. Mixed different surgeries cause large clinical heterogeneity. In comparison, the current meta-analysis focused on THA only.

### Implications for clinical practice

Our meta-analysis showed that the pain benefit existed in FICB compared with placebo. As we know, the femoral nerve and the lateral femoral cutaneous nerve innervate with the hip joint. FICB is injected through the iliac fascia cavity, which is constructed with the fascia as prezone and the iliopsoas as posterior. FICB could significantly block the femoral nerve, lateral femoral cutaneous nerve, and obturator nerve. Thus, FICB could decrease the pain intensity after THA. This beneficial effect was identified by previous trials. Besides, FICB is easy to administer, as it only requires ultrasound guidance. Steenberg et al. [23] performed a systematic review and revealed that FICB is an effective and relatively safe supplement in the preoperative pain management of hip fracture patients. The main outcome was consistent with our conclusion. Considering the results of the current meta-analysis and published studies, we suggest the administration FICB for pain control for THA patients.

Moreover, FICB could significantly decrease the morphine consumption and morphine-related complication (occurrence of nausea). McGraw-Tatum et al. [24] identified that FICB required less overall total opioids than the control group. And FICB is a relatively safe anesthesia technique as the needle point is away from the femoral nerve, femoral artery, and femoral vein [25].

### Limitations

The present meta-analysis has some existing limitations that should be noted. First, only seven RCTs were included in this meta-analysis, and more high-quality studies are needed to confirm the above conclusions in the future. Second, patients were administered with different dose and regimes of FICB, which may lead to large heterogeneity. Third, the follow-up time in the included RCT was limited, and therefore, some adverse events may be underestimated. Fourth, a functional outcome is not performed due to the insufficiency of relevant data; future studies should observe the effects of FICB for hip function.

## Conclusion

Our meta-analysis suggested that FICB but not placebo significantly reduced postoperative pain for THA patients. The use of FICB significantly reduced morphine consumption and the risk of nausea. FICB is recommended as an adjunct to multimodal anesthesia for THA patients. Future studies should explore optimal strategies (including drug and volume) of FICB.

## Additional file

Additional file 1: Search strategies in PubMed (DOCX 14 kb)

## Abbreviations

CI: Confidence intervals
FICB: Fascia iliaca compartment block
NSAIDs: Non-steroidal antiinflammatory drugs
PRISMA: Preferred Reporting Items for Systematic Reviews and Meta-Analyses
RCTs: Randomized controlled trials
RR: Risk ratio
THA: Total hip arthroplasty
VAS: Visual analog scale
WMD: Weighted mean difference
