# Supplementary material for: The efficacy of fascia iliaca compartment block for pain control after total hip arthroplasty: a meta-analysis
Source: J Orthop Surg Res. 2019 Jan 25;14:33. doi: 10.1186/s13018-018-1053-1 (PMC6347785; doi:10.1186/s13018-018-1053-1)
Supplement: Supplementary file 1 — Search strategies in PubMed (DOCX 14 kb) [file 13018_2018_1053_MOESM1_ESM.docx]

Additional file 1: Search strategies in Pubmed.

((((((((((((((((((((((Total Hip Replacement) OR Total Hip Replacements) OR Replacements, Total Hip) OR Hip Replacements, Total) OR Replacement, Total Hip) OR Hip Replacement, Total) OR Hip Replacement Arthroplasties) OR Arthroplasties, Hip Replacement) OR Replacement Arthroplasty, Hip) OR Replacement Arthroplasties, Hip) OR Hip Replacement Arthroplasty) OR Prosthesis Implantations, Hip) OR Prosthesis Implantation, Hip) OR Implantations, Hip Prosthesis) OR Implantation, Hip Prosthesis) OR Hip Prosthesis Implantations) OR Hip Prosthesis Implantation) OR Arthroplasty, Hip Replacement) OR Arthroplasties, Replacement, Hip) OR THA) OR total hip replacement) OR total hip arthroplasty) OR "Arthroplasty, Replacement, Hip"[Mesh] And (((fascia iliaca block) OR fascia iliaca compartment block)).
